# Evaluation of knowledge, attitude and practices towards loiasis in the rural community of Sindara, in central African Gabon

**DOI:** 10.1371/journal.pntd.0012109

**Published:** 2024-05-23

**Authors:** Teite Rebecca Hildebrandt, Saskia Dede Davi, Anita Lumeka Kabwende, Lilian Rene Endamne, Esther Mehmel, Maximilian Rakotonirinalalao, Ayodele Alabi, Rella Zoleko Manego, Peter G. Kremsner, Bertrand Lell, Ayôla Akim Adegnika, Ghyslain Mombo-Ngoma, Johannes Mischlinger, Selidji Todagbe Agnandji, Michael Ramharter

**Affiliations:** 1 Center for Tropical Medicine, Bernhard-Nocht Institute for Tropical Medicine and I. Department of Medicine University Medical Centre Hamburg-Eppendorf, Hamburg, Germany; 2 Centre de Recherches Médicales de Lambaréné, Lambaréné, Gabon; 3 German Center for Infection Research, Partner Sites Hamburg-Lübeck-Borstel-Riems, Hamburg, Germany; 4 Institut für Tropenmedizin, Eberhard-Karls-Universität Tübingen, Tübingen, Germany; 5 Department of Parasitology, Leiden University Medical Center, Leiden, The Netherlands; 6 Medical University of Vienna, Vienna, Austria; 7 German Center for Infection Research, Partner Site Tübingen, Germany; 8 Department of Implementation Research, Bernhard Nocht Institute for Tropical Medicine, Hamburg, Germany; Baylor College of Medicine, UNITED STATES

## Abstract

**Background:**

More than 20 million people are infected with *L*. *loa*, and around 40 million live in high or intermediate-risk areas in West- and Central Africa. Although loiasis is associated with significant morbidity and excess mortality, little is known about the perception of loiasis by affected communities. This study assessed the knowledge, attitudes, and practices in the rural population of Sindara, Gabon, a region characterized by high loiasis prevalence.

**Methods:**

A community-based cross-sectional survey was conducted in Gabon between January and June 2022. During systematic door-to-door visits, randomly selected inhabitants were invited to participate in this questionnaire based survey. Venous blood was collected at midday from all participants for microscopic detection of filarial infection and clinical signs of loiasis were assessed.

**Results:**

A total of 150 participants were recruited, of which 66% were infected by *L*. *loa*. While almost everyone had some knowledge about *L*. *loa*, 72% of the participants understood that *L*. *loa* is a parasitic worm. The transmission of *L*. *loa* via the deer fly was known to only 21% of participants. The most frequently mentioned clinical symptoms attributed to loiasis were itching (84%), eye worm migration (59%), and conjunctivitis-like symptoms (53%). Participants who experienced migratory loiasis had better knowledge of loiasis and considered it as more serious. Traditional and herbal medicine was reported most often as an available treatment option (72%). While the formal healthcare sector was mentioned as the preferred treatment provider, 60% of the reported infections were treated by traditional medical practitioners.

**Conclusion:**

Loiasis is in general well known by this community residing in a region of high *L*. *loa* transmission. Important gaps in knowledge were discovered foremost regarding the mode of transmission. The available healthcare system does not seem to provide adequate management for loiasis.

## Author summary

Loiasis is a filarial disease highly prevalent in parts of Western- and Central Africa. For a long time, the disease has been considered to be a relatively benign condition, but recent studies have shown that loiasis causes significant morbidity and excess mortality. In the present study, we investigated the perception of loiasis in a highly affected community in rural Gabon to better understand the community perspective on this infectious disease. While *L*. *loa* as a disease is known to most inhabitants, the mode of transmission and prevention modalities are only poorly understood. This lack of knowledge leaves the communities with inadequate means to prevent onward transmission of this filarial disease. The contribution from media, schools and healthcare institutions in providing information about loiasis is inadequate, and the available healthcare system is insufficient to provide the necessary care for loiasis. A better understanding of loiasis by the affected communities is desirable to empower inhabitants of high-transmission regions to better protect themselves from loiasis. Improved understanding of the disease by the healthcare sector is necessary to improve the management of loiasis.

## 1. Introduction

Loiasis is a vector-borne disease caused by the parasitic roundworm *Loa loa*. The parasite is transmitted by day-biting tabanid *Chrysops* flies and is restricted mainly to remote forest and savannah areas of West- and Central Africa [1]. More than 20 million individuals are infected with *L*. *loa*, and around 40 million people live in high or intermediate-risk areas with an estimated prevalence of eye worms of 40% or above [2]. Many affected communities live in hard-to-reach rural regions in the Central- and West African rainforest. Loiasis is particularly highly prevalent in Cameroon, Equatorial Guinea, Gabon, and parts of the Democratic Republic of Congo, the Central African Republic, and the Republic of Congo [2].

Adult parasites live under the skin and in the intermuscular fascia, while the microfilaria circulate in peripheral blood with a diurnal periodicity. Adult worms can live up to 20 years in their host, producing large numbers of microfilariae [3].

At times, the adult worm typically migrates through the subconjunctival tissue of the eye, leading to the common name “*African Eyeworm*”. Loiasis may present asymptomatically or with a wide range of symptoms, including Calabar swellings (itchy swellings mainly of the joints of arms and legs) and pruritus. Severe and life-threatening cerebral, renal, cardiac, and pulmonary complications have been reported [4].

Loiasis has been described as one of the most common reasons for medical consultations in high-prevalence regions. Quantification of the overall burden of disease caused by loiasis resulted in about 400 disability-adjusted life years (DALYs) per 100,000 people in rural Gabon. Hence, loiasis severely impairs affected individuals and is associated with significant morbidity comparable to that of other neglected tropical parasitic diseases [5]. Furthermore, it was shown that high-level *L*. *loa* microfilaraemia was associated with increased mortality. Microfilaraemia of more than 30,000 microfilaria per mL led to premature death compared to amicrofilaraemia. The population-attributable fraction of mortality associated with *L*. *loa* infection was 15% [6].

The infection was historically considered as relatively harmless and benign, but the recent evidence of high loiasis-attributable morbidity and mortality reveals the need for more medical research, treatment programs or control efforts for loiasis. Unlike onchocerciasis and lymphatic filariasis, loiasis is currently not included in the WHO’s list of neglected tropical diseases. Importantly, despite the high prevalence of loiasis in rural regions and its associated morbidity and mortality, there is an absence of national control programs for loiasis in Gabon [7,8].

While there remains an important mismatch between the scientific evidence for loiasis-related morbidity and mortality and the neglect of the disease by the national and international public health community, little is known about the knowledge and perceptions of affected communities living in high transmission regions of loiasis. Knowledge, attitudes, and practices are known determinants for investment in prevention, control, and management of diseases both on an individual level as well as on a programmatic scale [9,10]. Appreciating the perspective of affected communities is therefore essential to understand the perspective of the patients and to inform public health policy. To close existing gaps, this study assessed the knowledge, attitudes, and practices of a community in central Gabon characterized by a high prevalence of loiasis, towards the African eyeworm.

## 2. Material and Methods

### 2.1 Ethics statement

The study was part of an epidemiological study on infectious diseases (Demographic and Health Surveillance System/ “Identify diseases, undertake health research and provide better care for the Sindara population and nearby”) that was approved by the Institutional Ethics Committee of the Centre de Recherches Médicales de Lambaréné (CERMEL; Submission number: CEI-008/2021). Before the conduct of the study, the protocol was presented to community leaders in the study region to obtain their authorization to conduct the study in the community. Participation in the study was voluntary, and each participant provided written informed consent. For those who had no formal education the consent form was read out loud in the presence of an impartial witness (a close family member), providing time and opportunity for further clarifications and documentation by signing the informed consent. All data collected were anonymized to ensure confidentiality.

### 2.2 Study design and region

Between January and June 2022, a community-based cross-sectional survey was conducted in Sindara, a village in the tropical rainforest. Sindara is located in the department Tsamba-Magotsi in the province Ngounié in Southern-central Gabon, about 300 km southeast of the capital Libreville. The climate is equatorial, with two rainy seasons occurring from September to November and February to May. The average temperature is 25.9°C, and the humidity is usually over 80% [11]. The region around Sindara is characterized by dense tropical rainforest and the Ngounié River flowing through the village. There are two primary schools and a small dispensary for minor health issues in Sindara. Residents need to travel to the closest cities Fougamou (30km), Mouila (130 km), or Lambaréné (90 km), for higher-level education and healthcare facilities. Inhabitants of Sindara earn their livings primarily by hunting, fishing, subsistence farming in the surrounding rainforest, and employment in the local forestry industry. Large parts of Gabon, including the province Ngounié, are classified as high-transmission regions for loiasis, where the estimated prevalence of history of eye-worm migration ranges between 40% and 60% [2]. A previous study conducted in the Ngounie province showed that frequent forest exposure of people living in this region is a significant risk factor for loiasis [12]. Besides loiasis, the filarial disease mansonellosis, urogenital schistosomiasis, malaria, and infections by soil-transmitted helminths are highly prevalent. Malaria is the most frequent cause of health care attendance in Tsamba-Magotsi with a *Plasmodium spp*. infection prevalence of 37% [13]. Despite infrastructural deficits and considerable exposure to infectious diseases, local communities being engaged in subsistence farming and hunting activities for centuries, are long-term residents in these economically underprivileged regions of Gabon.

### 2.3 Study population and sampling strategy

To ensure unbiased representativeness of the population invited to this survey, the study activities were embedded in an ongoing demographic surveillance system of the region. In January 2022, a first round of a general census and mapping of all households and their members in Sindara was performed as part of this Demographic and Health Surveillance System. The census was done by trained fieldworkers using the mapping program QField and interviewing all inhabitants regarding their household members. In total, 786 buildings were identified, of which 356 (45%) were categorized as inhabited residential buildings. Out of these, 150 (42%) households were randomly selected to obtain a representative sample size of this community. Systematic door-to-door visits of the selected households were conducted. All inhabitants were screened for eligibility by dedicated study personnel. Eligibility criteria were age of >18 years, residence in Sindara for at least the past six months, and willingness to participate in the study. From each household, one person responding to the inclusion criteria of the survey was randomly selected by rolling dice. One hundred twenty-two participants were recruited during these visits at their homes. Due to repeated absence of the remaining 28 inhabitants during our visits, another convenience sample of 28 persons residing in the community were invited to participate in the survey to obtain the final sample size of 150 participants.

### 2.4 Procedure

#### Questionnaire-based interviews

Questionnaire-based interviews were conducted by a single, trained interviewer. The interviews were held individually and in privacy to avoid any bias introduced by the presence of others. The interview was conducted in French, the lingua franca in Gabon. Translations to local languages were performed by fieldworkers for those participants who were not fully capable of communication in French. The questionnaire-based interview was conducted as open questions to allow for unbiased answers. The answers were then coded in prespecified multiple-choice answer categories. Likert scales were used to capture the attitude towards loiasis semi-quantitatively.

The survey was divided into six main sections with 39 questions. The questions focused on the following topics: i) socio-demographic characteristics (gender, age, education); ii) knowledge about loiasis*-*related signs and symptoms, transmission, prevention and treatment; iii) attitudes towards the seriousness of loiasis, the risk of infection and the presence of stigma associated with the infection; iv) practices regarding lifestyle, prevention of loiasis and healthcare seeking behavior; v) personal history of loiasis, including RAPLOA, the rapid assessment method of the history of eye worm migration endorsed by the WHO [14]. The questionnaire was created with contributions from disease experts, patients, and community representatives. Pretesting was performed to validate the questionnaire in Gabonese individuals not residing in Sindara.

#### Parasitological examinations

This survey was designed to evaluate primarily the KAP of the general population residing in a high transmission region of Gabon. However, pre-defined secondary outcomes included the exploratory analysis of potential differences of KAP between infected and uninfected residents and types of infection, respectively. For this purpose a parasitological examination was performed in parallel to the survey. However, participants were not included or excluded based on the parasitological test results. Venous blood was collected between 10 am and 3 pm in EDTA tubes coinciding with the peak microfilaraemia of *L*. *loa*. Tubes were stored in the dark at 4°C (± 1°C) before transportation to the laboratory. Microfilaria detection was performed by leuco-concentration-technique (1 ml of blood; 1 ml of 2% saponin-solution) [15]. Infection with *L*. *loa* was defined as either presence of *L*. *loa* microfilariae in the blood sample or a positive history of eyeworm migration. An additional malaria rapid test was performed, and first-line antimalarial therapy was offered to those participants with positive test results.

### 2.5 Data analysis

Data were managed using REDCap electronic data capture tools hosted at CERMEL and transferred for further statistical analysis (Stata/SE 16.1, StataCorp, College Station, USA). For descriptive analysis, the absolute frequency and the percentages were computed to express the proportion of variable categories. The 95% confidence interval was used as a measure of the precision of the obtained population means.

## 3. Results

Out of 150 participants, 71 (47%) were female, and 79 (53%) were male. The age ranged between 19 and 85 years, with a median age of 45.5 (IQR: 32–59). Eleven participants (8%) reported no school attendance, 68 (45%) completed one to six years, and 70 (47%) more than six years of school attendance (Table 1).

Among all participants, 99 (66%) were defined as *L*. *loa* positive, either by detecting microfilariae in peripheral blood or having a positive history of eyeworm migration (RAPLOA). 51 participants (34%) were defined as *L*. *loa* negative by the absence of microfilaria and a negative history of eye worm migration.

**Table 1:** Demographics of the study population.

Demographics (N = 150)	n (col %)
**Gender**	
Female	71 (47)
Male	79 (53)
**Age**	
19–30	35 (23)
31–40	26 (17)
41–50	33 (22)
50+	56 (37)
**Educational level**	
No education	11 (7)
6 years or less (≤6 years)	68 (45)
more than 6 years (>6 years)	71 (47)

n = number; col = column; % = percentage.

### 3.1 Knowledge

In Sindara *L*. *loa* is mainly known under the term “*Doba*” (in the local Gisir language) or “*Hogho*” (in local Getsogo language), two of the most widely spoken Bantu languages in this region. Results regarding knowledge towards *L*. *loa* are shown in Table 2.

#### Disease presentation and burden

Almost every respondent had heard of the pathogen *L*. *loa* (n = 149; 99%). Fewer participants knew that *L*. *loa* is a parasitic worm (n = 108; 72%). Others referred to it as bacteria or virus (n = 10; 7%) or had no notion of which group of pathogens *L*. *loa* belongs to (n = 28; 19%). By far, the most frequently mentioned symptoms attributed to *loiasis* were itching (n = 126; 84%), eye worm migration (n = 88; 59%), conjunctivitis, eye congestion, pain and light sensitivity (n = 80; 53%), urticaria (n = 53; 35%) and transient swelling of parts of arms and legs (n = 51; 34%). Less frequently mentioned symptoms were transient vision loss (n = 29; 19%), transient worm migration on other parts of the body than the eye (n = 26; 17%), muscle and joint pain (n = 10; 7%), fatigue (n = 5; 3%) and transient paralysis (n = 1; 1%). In addition, 19% (n = 29) mentioned other symptoms, including mainly headache and fever. 3% (n = 4) did not know any sign or symptom of loiasis. Half of the participants stated that signs and symptoms of loiasis sometimes prevent them from working and doing household chores.

#### Transmission

The majority of participants did not know the correct mode of transmission of *L*. *loa* (n = 78; 52%) or indicated incorrect modes of transmission, such as water and food (n = 30; 20%), mosquitoes (n = 10; 7%), human to human (n = 4; 3%), animals (n = 1; 1%) and other sources of infection (n = 18; 12%). One-fifth (n = 31; 21%) correctly answered *Chrysops* (locally called “la mouche rouge”) as the mode of transmission of *L*. *loa*. Interestingly, most people correctly answered questions about the biting habits of *Chrysops* spp. (n = 145; 97% daytime) and the period of the highest activity of *Chrysops* (n = 102; 68% rainy season), indicating high levels of knowledge about *Chrysops* as a general nuisance in this region.

#### Prevention

27% (n = 41) of the participants stated that loiasis cannot be prevented, and 44% (n = 66) indicated that they do not know how to prevent loiasis. Besides this, the use of long-sleeved clothes and pants (n = 18; 12%), the use of mosquito nets during the day (n = 11; 7%), the use of insect repellants (n = 1; 1%), medical prevention with diethlcarbamazine (n = 1, 1%) and others (n = 19, 13%) were mentioned.

#### Treatment

The majority of participants (n = 108; 72%) indicated the existence of traditional and herbal medicines as treatment options for loiasis. A further 47% (n = 70) mentioned anthelminthic drugs, and 13% (n = 19) the surgical removal of the adult worm during the migration through the eye as treatment modality. Treatment was considered to be relatively well accessible (n = 121; 81%), although this referred mainly to traditional treatment (n = 91; 61%) and less so for the formal healthcare sector (n = 44; 29% hospitals, 3% (n = 5) dispensary, 3% (n = 5) pharmacy, 1% (n = 2) at supermarkets).

#### Source of information

The most frequent and almost unique source of information for the above-mentioned topics were neighbors, friends and family (n = 144; 96%). Only 14% (n = 20) of the participants learned about *L*. *loa* at school (n = 10; 7%) or at a health center (n = 10; 7%).

**Table 2:** Knowledge of *Loa loa* by local residents.

Knowledge	Overall (N = 150)		*L*. *loa* negative (N = 51)		*L*. *loa* positive (N = 99)			
					Non-migratory (N = 7)		Migratory (N = 92)	
	n (col %)	95% CI	n (col %)	95% CI	n (col %)	95% CI	n (col %)	95% CI
**Have you heard about *L*. *loa*?**								
Yes	149 (99)	[96; 100]	50 (98)	[90; 100]	7 (100)	[59; 100]*	92 (100)	[96; 100]▴
No	1 (1)	[0; 4]	1 (2)	[0; 10]	0 (0)		0 (0)	
**What is *L*. *loa*?**								
Worm	108 (72)	[64; 79]	26 (51)	[37; 65]	4 (57)	[18; 90]	78 (85)	[76; 91]
Other	4 (3)	[1; 7]	2 (4)	[0; 13]	0 (0)		2 (2)	[0; 8]
Bacteria, Virus	10 (7)	[3; 12]	6 (12)	[4; 24]	2 (29)	[4; 71]	2 (2)	[0; 8]
Don’t know	28 (19)	[13; 26]	17 (33)	[21; 48]	1 (14)	[0; 58]	10 (11)	[5; 19]
**What are signs and symptoms of loiasis?** *								
Transient swelling of parts of arms/legs	51 (34)	[26; 42]	17 (33)	[21; 48]	3 (43)	[10; 82]	31 (34)	[24; 44]
Itching	126 (84)	[77; 89]	41 (80)	[67; 90]	7 (100)	[59; 100]▴	78 (85)	[76; 91]
Eyeworm migration	88 (59)	[50; 67]	21 (41)	[28; 56]	3 (43)	[10; 82]	64 (70)	[59; 79]
Conjunctivitis, eye congestion, itching, pain, light sensitivity	80 (53)	[45; 62]	16 (31)	[19; 46]	2 (29)	[4; 71]	62 (67)	[57; 77]
Transient vision loss	29 (19)	[13; 27]	4 (8)	[2; 19]	1 (14)	[0; 58]	24 (26)	[17; 36]
Transient worm migration on other parts of the body than eye	26 (17)	[12; 24]	3 (6)	[1; 16]	2 (29)	[4; 71]	21 (23)	[15; 33]
Hives	53 (35)	[28; 44]	17 (33)	[21; 48]	4 (57)	[18; 90]	32 (35)	[25; 45]
Muscle pain/ Joint pain	10 (7)	[3; 12]	1 (2)	[0; 10]	0 ()		9 (10)	[5; 18]
Tiredness	5 (3)	[1; 8]	2 (4)	[1; 13]	1 (14)	[0; 58]	2 (2)	[0; 8]
Toothache	27 (18)	[12; 25]	5 (10)	[3; 21]	0 (0)		22 (24)	[16; 34]
Transient paralysis	1 (1)	[0; 4]	0 (0)		0 (0)		1 (1)	[0; 6]
Other	29 (19)	[13; 27]	6 (12)	[4; 24]	1 (14)	[0; 58]	22 (24)	[16; 34]
Don’t know	4 (3)	[1; 7]	4 (8)	[2; 19]	0 (0)		0 (0)	
**Does loiasis prevent you from working/ household chores?**								
Yes	75 (50)	[42; 58]	20 (39)	[26; 54]	2 (29)	[4; 71]	53 (58)	[47; 68]
No	68 (45)	[37; 54]	25 (49)	[35; 63]	5 (71)	[29; 96]	38 (41)	[31; 52]
Don’t know	7 (5)	[2; 9]	6 (12)	[4; 24]	0 (0)		1 (1)	[0; 6]
**How do people get infected?** *								
Fly. or deer fly. or *Chrysops* (“the red fly”)	31 (21)	[14; 28]	6 (12)	[4; 24]	1 (14)	[0; 58]	24 (26)	[17; 36]
Mosquitoes	10 (7)	[3; 12]	4 (8)	[2; 19]	0 (0)		6 (7)	[2; 14]
Water. food	30 (20)	[14; 27]	10 (20)	[10; 33]	1 (14)	[0; 58]	19 (21)	[13; 30]
Human-human	4 (3)	[1; 7]	3 (6)	[1; 16]	0 (0)		1 (1)	[0; 6]
Animals	1 (1)	[0; 4]	0 (0)		0 (0)		1 (1)	[0; 6]
Mystique	(0)		0 (0)		0 (0)		0 (0)	
Other	18 (12)	[7; 18]	5 (10)	[3; 21]	2 (29)	[4; 71]	11 (12)	[6; 20]
Don’t know	78 (52)	[44; 60]	30 (59)	[44; 72]	3 (43)	[10; 82]	45 (49)	[38; 60]
**How can you prevent loiasis?** *								
Cannot be prevented	41 (27)	[20; 35]	11 (22)	[11; 35]	0 (0)		30 (33)	[23; 43]
Avoiding areas where *Chrysops* live/ brood / take blood meals	0 (0)		0 (0)		0 (0)		0 (0)	
Using insect repellents	1 (1)	[0; 4]	1 (2)	[0; 10]	0 (0)		0 (0)	
Using mosquito coils	0 (0)		0 (0)		0 (0)		0 (0)	
Wearing long sleeves/ long pants during the day	18 (12)	[7; 18]	4 (8)	[2; 19]	3 (43)	[10; 82]	11 (12)	[6; 20]
Rest under a Mosquito net during the day	11 (7)	[4; 13]	5 (10)	[3; 21]	0 (0)		6 (7)	[2; 14]
Medical prevention (Diethylcarbamazine 300mg/week)	1 (1)	[0; 4]	0 (0)		0 (0)		1 (1)	[0; 6]
Other	19 (13)	[8; 19]	9 (18)	[8; 31]	0 (0)		10 (11)	[5; 19]
Don’t know	66 (44)	[36; 52]	25 (49)	[35; 63]	4 (57)	[18; 90]	37 (40)	[30; 51]
**Is there a treatment for loiasis?** *								
Yes	136 (91)	[85; 95]	43 (84)	[71; 93]	7 (100)	[59; 100]▴	86 (93)	[86; 98]
No	2 (1)	[0; 5]	1 (2)	[0; 10]	0 (0)		1 (1)	[0; 6]
Don’t know	12 (8)	[4; 14]	7 (14)	[6; 26]	0 (0)		5 (5)	[2; 12]
If yes, please specify:								
Drugs	70 (47)	[38; 55]	23 (45)	[31; 60]	4 (57)	[18; 90]	43 (47)	[36; 57]
Operation	19 (13)	[8; 19]	7 (14)	[6; 26]	0 (0)		12 (13)	[7; 22]
Traditional/ Herbal medicine	108 (72)	[64; 79]	32 (63)	[48; 76]	6 (86)	[42; 100]	70 (76)	[66; 84]
Other	1 (1)	[0; 4	1 (2)	[0; 10]	0 (0)		0 (0)	
**Is the treatment available for you if you have loiasis?** *								
Yes	121 (81)	[73; 87]	34 (67)	[52; 79]	7 (100)	[59; 100]▴	80 (87)	[78; 93]
No	17 (11)	[7; 18]	10 (20)	[10; 33]	0 (0)		7 (8)	[3; 15]
Don’t know	12 (8)	[4; 14]	7 (14)	[6; 26]	0 (0)		5 (5)	[2; 12]
If yes, please specify:								
Hospital	44 (29)	[22; 37]	12 (24)	[13; 37]	4 (57)	[18; 90]	28 (30)	[21; 41]
Dispensary	5 (3)	[1; 8]	0 (0)		0 (0)		5 (5)	[2; 12]
Pharmacy	5 (3)	[1; 8]	2 (4)	[0; 13]	0 (0)		3 (3)	[1; 9]
Traditional Healer	91 (61)	[52; 69]	26 (51)	[37; 65]	6 (86)	[42; 100]	59 (64)	[53; 74]
Supermarket	2 (1)	[0; 5]	1 (2)	[0; 10]	0 (0)		1 (1)	[0; 6]
If no, please specify:								
Not available	11 (7)	[4; 13]	6 (12)	[4; 24]	0 (0)		5 (5)	[2; 12]
Too costly	3 (2)	[0; 6]	2 (4)	[0; 13]	0 (0)		1 (1)	[0; 6]
Too far away	5 (3)	[1; 8]	3 (6)	[1; 16]	0 (0)		2 (2)	[0; 8]
Other	0 (0)		0 (0)		0 (0)		0 (0)	
**Deer fly behaviour**								
**Do people get bitten more likely during the day or during the night?**								
Daytime	145 (97)	[92; 99]	48 (94)	[84; 99]	7 (100)	[59; 100]▴	90 (98)	[92; 100]
Nighttime	1 (1)	[0; 4]	1 (2)	[0; 10]	0 (0)		0 (0)	
Any time	4 (3)	[1; 7]	2 (4)	[0; 13]	0 (0)		2 (2)	[0; 8]
**In which season does the vector mostly appear?**								
Rainy season	102 (68)	[60; 75]	29 (57)	[42; 71]	5 (71)	[29; 96]	68 (74)	[64; 83]
Dry season	15 (10)	[6; 16]	7 (14)	[6; 26]	0 (0)		8 (9)	[4; 16]
Any season	31 (21)	[14; 28]	14 (27)	[16; 42]	2 (29)	[4; 71]	15 (16)	[9; 25]
Don’t know	2 (1)	[0; 5]	1 (2)	[0; 10]	0 (0)		1 (1)	[0; 6]
**Source of information**								
**Where do you get your information?** *								
Health clinic/ Hospital	10 (7)	[3; 12]	2 (4)	[0; 13]	2 (29)	[4; 71]	6 (7)	[2; 14]
Dispensary	4 (3)	[1; 7]	1 (2)	[0; 10]	0 (0)		3 (3)	[1; 9]
Media	1 (1)	[0; 4]	1 (2)	[0; 10]	0 (0)		0 (0)	
School	10 (7)	[3; 12]	2 (4)	[0; 13]	0 (0)		8 (9)	[4; 16]
Neighbours, Friends, Family	144 (96)	[91; 99]	48 (94)	[84; 99]	7 (100)	[59; 100]▴	89 (97)	[91; 99]
Church	0 (0)		0 (0)		0 (0)		0 (0)	
Traditional healer	2 (1)	[0; 5]	1 (2)	[0; 10]	0 (0)		1 (1)	[0; 6]
Other	1 (1)	[0; 4]	0 (0)		0 (0)		1 (1)	[0; 6]
Do not remember	2 (1)	[0; 5]	2 (4)	[0; 13]	0 (0)		0 (0)	

n = number; col = column; % = percentage

* = multiple response question

95% CI = 95% Confidence interval

▴ = 97.5% Convidence interval.

### 3.2 Attitudes

The seriousness of *loiasis* was rated very diversely by the participants (Table 3). The mean on a Likert scale from 1 –not serious to 5 –very serious was at 3.3. Regarding the respondent’s own risk perception (1 –no risk to 5 –serious risk) the results were distributed similarly with a mean of 3.4. Concerning the question on how many people are affected by loiasis (1- no one to 5- everyone is infected), people were aware that loiasis probably affects many residents of Sindara as indicated by a mean of 3.1. The majority of participants stated that there exists no stigma or social exclusion towards people suffering from loiasis (n = 143; 95%).

**Table 3:** Attitudes on *Loa loa* by local residents.

Attitude	Overall		*L*. *loa* negative		L. loa positive			
					Non–migratory		Migratory	
	Mean	95% CI	Mean	95% CI	Mean	95% CI	Mean	95% CI
**Is Loiasis a serious disease?**	**(N = 145)**		**(N = 47)**		**(N = 7)**		**(N = 91)**	
1 not serious– 5 very serious	3.3	[3.1; 3.5]	3.2	[2.9; 3.5]	2.4	[1.4–3.5]	3.4	[3.1–3.7]
2								
3 neutral								
4								
5 very serious								
**Are you at risk of getting infected with loiasis?**	**(N = 137)**		**(N = 47)**		**(N = 7)**		**(N = 83)**	
1 no risk	3.4	[3.1; 3.6]	3	[2.6; 3.5]	2.9	[1.2–4.5]	3.6	[3.3–3.9]
2								
3 neutral								
4								
5 serious risk								
**Do you think many people in Sindara suffer from loiasis?**	**(N = 134)**		**(N = 45)**		**(N = 6)**		**(N = 83)**	
1 no one	3.1	[2.9; 3.3]	2.7	[2.4; 3.0]	2.5	[1.6–3.4]	3.4	[3.2–3.6]
2								
3 neutral								
4								
5 everyone								
	**n (col %)**	**95% CI**	**n (col %)**	**95% CI**	**n (col %)**	**95% CI**	**n (col %)**	**95% CI**
**Have you ever experienced exclusion and stigma towards a person suffering from loiasis?**	**(N = 150)**		**(N = 51)**		**(N = 7)**		**(N = 92)**	
Yes	5 (3)	[1; 8]	1 (2)	[0; 10]	0 (0)		4 (4)	[1; 11]
No	143 (95)	[91; 98]	49 (96)	[87; 100]	7 (100)	[59; 100]▴	87 (95)	[88; 98]
Don‘t know	2 (1)	[0; 5]	1 (2)	[0; 10]	0 (0)		1 (1)	[0; 6]

n = Number; col = column; % = percentage; 95% CI = 95% Confidence interval

▴ = 97.5% Confidence interval

### 3.3 Practices

About one-third of participants reported sleeping outside during the day from time to time (n = 52; 35%). While most of the participants prepared their food inside a closed house or shelter (n = 109; 73%), one-fourth also prepared in unclosed houses or shelters (n = 37; 25%) and a few prepared outdoors over a woodfire (n = 7; 5%). The majority stated that they wash their clothes at the river (n = 136; 91%), and more than two-thirds worked in the forest regularly (n = 101; 67%). Nonetheless, 47% (n = 71) stated that they do not actively protect themselves from loiasis, and 49% (n = 73) indicated wearing long sleeves and pants during the day. Regarding the health-seeking behaviour, the largest part of people indicated preferring consultation at a hospital in case of loiasis-related symptoms (n = 60; 40%), followed by self-treatment (n = 39; 26%) and traditional healers (n = 31; 21%; Table 4).

**Table 4:** Practices regarding *Loa loa* by local residents.

Practise	Overall (N = 150)		*L*. *loa* negative (N = 51)		*L. loa* positive (N = 99)			
					Non—migratory (N = 7)		Migratory (N = 92)	
	n (col %)	95% CI	n (col %)	95% CI	n (col %)	95% CI	n (col %)	95% CI
**Do you sleep outside during the day?**								
Yes	52 (35)	[27; 43]	19 (37)	[24; 52]	2 (29)	[4; 71]	31 (34)	[24; 44]
No	98 (65)	[57; 73]	32 (63)	[48; 76]	5 (71)	[29; 96]	61 (66)	[56; 76]
**Where do you prepare your food?** *								
Inside a house or shelter	109 (73)	[65; 80]	39 (76)	[63; 87]	4 (57)	[18; 90]	66 (72)	[61; 81]
House or shelter that isn’t closed	37 (25)	[18; 32]	11 (22)	[11; 35]	2 (29)	[4; 71]	24 (26)	[17; 36]
Outside	7 (5)	[2; 9]	2 (4)	[0; 13]	0 (0)		5 (5)	[2; 12]
Other	2 (1)	[0; 5]	0 (0)		1 (14)	[0; 58]	1 (1)	[0; 6]
**Where do you wash your clothes?** *								
At home	13 (9)	[5; 14]	6 (12)	[4; 24]	0 (0)		7 (8)	[3; 15]
River	136 (91)	[85; 95]	46 (90)	[79; 97]	6 (86)	[42; 100]	84 (91)	[84; 96]
Other	10 (7)	[3; 12]	3 (6)	[1; 16]	1 (14)	[0; 58]	6 (7)	[2; 14]
**Do you work regularly in the forest?**								
Yes	101 (67)	[59; 75]	30 (59)	[44; 72]	5 (71)	[29; 96]	66 (72)	[61; 81]
No	49 (33)	[25; 41]	21 (41)	[28; 56]	2 (29)	[4; 71]	26 (28)	[19; 39]
**Do you prevent yourself from loiasis?–** **What do you do?** *								
Avoiding areas where *Chrysops* live/ brood / take blood meals	0 (0)		0 (0)		0 (0)		0 (0)	
Using insect repellents	0 (0)		0 (0)		0 (0)		0 (0)	
Using mosquito coils	0 (0)		0 (0)		0 (0)		0 (0)	
Wearing long sleeves/ long pants during the day	73 (49)	[40; 57]	23 (45)	[31; 60]	5 (71)	[29; 96]	45 (49)	[38; 60]
Rest under a Mosquito net during the day	6 (4)	[1; 9]	5 (10)	[3; 21]	0 (0)		1 (1)	[0; 6]
Medical prevention (Diethylcarbamazine 300mg/week)	0 (0)		0 (0)		0 (0)		0 (0)	
Other	11 (7)	[4; 13]	6 (12)	[4; 24]	0 (0)		5 (5)	[2; 12]
Do not prevent	71 (47)	[39; 56]	26 (51)	[37; 65]	2 (29)	[4; 71]	43 (47)	[36; 57]
**Health seeking behaviour**								
**If you had symptoms of loiasis, where would you look for help first?** *								
Hospital	60 (40)	[32; 48]	26 (51)	[37; 65]	2 (29)	[4; 71]	32 (35)	[25; 45]
Dispensary	12 (8)	[4; 14]	2 (4)	[0; 13]	1 (14)	[0; 58]	9 (10)	[5; 18]
Pharmacy	2 (1)	[0; 5]	2 (4)	[0; 13]	0 (0)		0 (0)	
Traditional Healer	31 (21)	[14; 28]	9 (18)	[8; 31]	3 (43)	[10; 82]	19 (21)	[13; 30]
Self-Treatment	39 (26)	[19; 34]	8 (16)	[7; 29]	0 (0)		31 (34)	[24; 44]
Wait till it goes away	10 (7)	[3; 12]	5 (10)	[3; 21]	1 (14)	[0; 58]	4 (4)	[1; 11]
Other	4 (3)	[1; 7]	0 (0)		0 (0)		4 (4)	[1; 11]
Don’t know	1 (1)	[0; 4]	1 (2)	[0; 10]	0 (0)		0 (0)	

n = Number; col = column; % = percentage

* = multiple response question

95% CI = 95% Convidence interval.

### 3.4 Individual experience

Almost all participants reported already having been bitten by *Chrysops* (n = 145; 97%), which according to the participants, mainly occurs in the forest and on subsistence farms near the village (Table 5). Additionally, 76% (n = 114) indicated that they suffer or suffered in the past from loiasis, or know someone who did (n = 41; 27%). Only 12% (n = 18) of the participants indicated not having been infected and not knowing someone infected with *L*. *loa*.

Interestingly, 60% (n = 90) of the participants reported that their *L*. *loa* infection was treated traditionally, while only 28% (n = 42) were treated with anthelminthic drugs, and 9% (n = 13) were not treated at all. The traditional treatment included primarily local administration of leaves and other spicy or acid extracts such as lemon, Costacea plant, vinegar, hot pepper sauce, onion, garlic and car fuel in the eye or on the skin when the adult worm appears during its migration. In addition, using barks and plants without further specification, or the direct removal of the adult worm using hooks or needles was reported as a treatment modality employed in Sindara.

**Table 5:** Individual experiences with *Loa loa* by local residents.

Own Experience	Overall (N = 150)		*L*. *loa* negative (N = 51)		*L. loa* positive (N = 99)			
					Non—migratory (N = 7)		Migratory (N = 29)	
	n (col %)	95% CI	n (col %)	95% CI	n (col %)	95% CI	n (col %)	95% CI
**Have you ever been bitten by a deer fly?**								
Yes	145 (97)	[92; 99]	47 (92)	[81; 98]	7 (100)	[59; 100]▴	91 (99)	[94; 100]
No	4 (3)	[1; 7]	4 (8)	[2; 19]	0 (0)		0 (0)	
Don’t know	1 (1)	[0; 4]	0 (0)		0 (0)		1 (1)	[0; 6]
**Where in Sindara does the deer fly appear most often?** *								
Forest	90 (60)	[52; 68]	29 (57)	[42; 71]	6 (86)	[42; 100]	55 (60)	[49; 70]
Plantation	80 (53)	[45; 62]	24 (47)	[33; 62]	6 (86)	[42; 100]	50 (54)	[44; 65]
In the village	69 (46)	[38; 54]	22 (43)	[29; 58]	2 (29)	[4; 71]	45 (49)	[38; 60]
Church	0 (0)		0 (0)		0 (0)		0 (0)	
Next to the river	29 (19)	[13; 27]	13 (25)	[14; 40]	3 (43)	[10; 82]	13 (14)	[8; 23]
Other	2 (1)	[0; 5]	1 (2)	[0; 10]	0 (0)		1 (1)	[0; 6]
Don’t know	1 (1)	[0; 4]	1 (2)	[0; 10]	0 (0)		0 (0)	
**Have you, or someone you know, been infected with loiasis?** *								
Yes, myself	114 (76)	[68; 83]	27 (53)	[38; 67]	5 (71)	[29; 96]	82 (89)	[81; 95]
Yes, someone I know	41 (27)	[20; 35]	17 (33)	[21; 48]	1 (14)	[0; 58]	23 (25)	[17; 35]
No	16 (11)	[6; 17]	10 (20)	[10; 33]	1 (14)	[0; 58]	5 (5)	[2; 12]
Don’t know	2 (1)	[0; 5]	1 (2)	[0; 10]	0 (0)		1 (1)	[0; 6]
**If yes, how was it treated?** *								
Not treated	13 (9)	[5; 14]	4 (8)	[2; 19]	0 (0)		9 (10)	[5; 18]
Drugs	42 (28)	[21; 36]	13 (25)	[14; 40]	4 (57)	[18; 90]	25 (27)	[18; 37]
Traditional	90 (60)	[52; 68]	26 (51)	[37; 65]	3 (43)	[10; 82]	61 (66)	[56; 76]
Don’t remember	3 (2)	[0; 6]	2 (4)	[0; 13]	0 (0)		1 (1)	[0; 6]
Not applicable	18 (12)	[7; 18]	11 (22)	[11; 35]	1 (14)	[0; 58]	6 (7)	[2; 14]

n = Number; col = column; % = percentage

* = multiple response question

95% CI = 95% Convidence interval

▴ = 97.5% Convidence interval.

### 3.5 Influence of infection status on KAP

A sub-analysis investigated the responses of persons with different infection states of *L*. *loa*. Here *L*. *loa*

positive participants were further grouped into patients with migratory (n = 92; 61%) or non-migratory loiasis (n = 7; 5%). Migratory loiasis is defined by a positive history of eyeworm migration (RAPLoa), regardless of whether microfilariae are found in peripheral blood. Participants with migratory loiasis had significantly better knowledge of the causal pathogen of loiasis as a worm (85%; 95% CI: 76 to 91%) compared to patients with non-migratory loiasis (57%; 95% CI: 18 to 90%) or uninfected participants (51%; 95% CI: 37 to 65%). Similarly, point estimates for the knowledge of transmission of *L*. *loa* were higher in migratory loiasis participants (26%; 95% CI: 17 to 36%) compared to non-migratory (14%; 95% CI: 0 to 58%) and uninfected participants (12%; 95% CI: 4 to 24%), although this difference was not statistically significant as evidenced by the overlapping 95% confidence intervals; participants suffering from migratory loiasis indicated more often that loiasis prevents them from working and performing household chores (58%; 95% CI: 47 to 68%) than non-migratory (39%; 95% CI: 26 to 54%) and uninfected (29%; 95% CI: 4 to 71%) participants.

Some signs and symptoms of loiasis were more commonly attributed to the disease by participants with migratory loiasis than by uninfected or non-migratory loiasis participants including eyeworm migration (70% migratory loiasis [95% CI: 59 to 79%] versus 41% uninfected [95% CI: 28 to 56%] and 43% non-migratory loiasis [95% CI: 10 to 82%]), conjunctivitis, eye congestion, itching, pain, light sensitivity (67% migratory loiasis [95% CI: 57 to 77%] versus 31% uninfected [95% CI: 19 to 46%], 29% non-migratory loiasis [95% CI: 4 to 71%]), and transient vision loss (26% migratory loiasis [95% CI: 17 to 36%] versus 8% uninfected [95% CI: 2 to 19%], 14% non-migratory loiasis [95% CI: 0 to 58%]).

Regarding the seriousness of loiasis (1—*not serious*—5—*very serious*) and the own risk assessment (1—no risk—5 -serious risk) differences were not statistically significant, even though migratory participants perceived the disease as worse (Mean: 3.4; 95% CI: 3.1 to 3.7 versus mean: 3.2; 95% CI: 2.9 to 3.5 and mean: 2.4; 95% CI: 1.4 to 3.5), and their own risk of getting infected as higher (Mean: 3.6; 95% CI: 3.3 to 3.9) than non-migratory loiasis patients (Mean: 3; 95% CI: 2.6 to 3.5) and non-infected participants (Mean: 2.9; 95% CI: 1.2 to 4.5).

## 4. Discussion

*L*. *loa* is highly prevalent in Gabon. In 2018, published data on the prevalence of *L*. *loa* infection in the Tsamba-Magotsi district revealed a prevalence of 31% by either the detection of microfilariae in the blood or a positive history of eyeworm migration, while the present study showed a prevalence of 66% using the same case definition [12]. This may indicate a higher overall exposure towards *L*. *loa* in the village Sindara due to its location deep in the tropical rainforest and next to the river Ngounié compared to the average settlements in Tsamba-Magotsi. Other data based on RAPLOA surveys conducted in 2011 all over Western and Central Africa estimated a prevalence of eyeworm migration between 40 and 60% in this area of Gabon. Our data confirm this high number and even exceed this estimation since it also includes asymptomatic microfilaraemic individuals.

Loiasis is well known in Sindara, almost everyone has heard of this disease and the majority further knew that the pathogen is a parasitic worm. The present survey was carried out in a region of high prevalence which may explain the high level of knowledge on *L*. *loa* by the population. This is also consistent with the finding that the vast majority declared to have been infected or to know someone infected by loiasis, as can be expected in an endemic region. Even though the red fly (*Chrysops*) and its biting habits were well known to almost every participant, its role as a vector in transmitting *L*. *loa* was widely unknown (80%). This is surprising given the high prevalence of loiasis and the overall good knowledge of both the disease and the presence of *Chrysops* flies in the region. However, our findings align with previously reported assessments of the KAP towards onchocerciasis, lymphatic filariasis and soil-transmitted helminths in Cameroon, showing a lack of knowledge, especially concerning the transmission of these neglected tropical diseases even though they were well-known and highly prevalent in the respective regions [16,17]. A similar example of poor knowledge of transmission modalities was reported for schistosomiasis in Yemen [18].

Information about loiasis was mainly passed on by word of mouth in Sindara. Our study revealed poor contribution from media, schools and healthcare facilities to the knowledge of loiasis. More attention should be paid to possible ways of distributing reliable and trustful information by patient education. It seems reasonable to assume that knowledge, especially about the transmission of the above-named diseases including loiasis could be an important and necessary mean for the development of effective prevention strategies.

In general, it was difficult for most participants to distinguish between specific diseases and their infectious etiologies. In general, many clinical presentations were generally assigned to "the worms" and no specific difference was made between filariae, soil-transmitted-helminths, scabies, and other parasitical diseases. This concept is reflected by the fact that participants often named water, food, and mosquitos, besides the *Chrysops* fly, as possible modes of transmission for loiasis. Similar patterns concerning the knowledge about signs and symptoms of loiasis were observed. Besides some specific symptoms including the eye worm migration and accompanying conjunctivitis, non-specific symptoms like pruritus and urticaria, which may be as well caused to multiple other parasitic diseases, were often mentioned. This carries also the risk that people living in these communities and positive for other chronic diseases might associate their symptoms with “the worms”, further delaying adequate healthcare seeking behavior, testing, and diagnosis.

Questions focusing on prevention of infection indicated that most participants reported that loiasis was not preventable, while others mentioned the use of long-sleeved shirts and pants and the use of mosquito nets during the day. However, these methods were not mentioned as prevention techniques specific for loiasis, but rather as a general protective measure during exposure to the surrounding rainforest. This goes in concordance with the current scientific evidence as little is known about effective prevention methods [12]. The importance of forest exposure, on the other hand, has been highlighted in several studies as a major epidemiological risk factor for the successful transmission of *L*. *loa*, while the absence of regular activities in the forest serves as a protective factor [12,19,20]. Reducing forest exposure may be a potential preventive measure which however remains difficult to implement since affected communities are often economically dependent on subsistence farming and hunting activities. Furthermore, the attraction of *Chrysops* by woodfires, which are commonly used for cooking in Sindara, indicates the difficulties in effectively preventing *Chrysops* bites in this environment [21].

The attitude of the participants towards loiasis, its severity and impact as well as the perception of the risk of getting infected in Sindara varied considerably. These differing attitudes reflect the wide range of clinical manifestations of the disease [22]. Importantly, a significant proportion of patients reported that loiasis at times prevents them from working providing evidence for the impact of the disease on daily activities. Interestingly, participants unanimously agreed on the fact that there is no stigma nor social exclusion regarding persons suffering from loiasis. This is an important outcome of this survey, although its external validity requires to be investigated in other cultural settings.

Although the formal healthcare system was cited as the preferred treatment provider, the majority of previous *L*. *loa* infections had been treated by traditional healers. This finding indicates a lack of medically adequate or economically accessible care provided by the formal health care system. While applying sour or acid extracts into the eye to chase the worm away or even to remove the parasite by fishhooks and needles reportedly soothed the patients, the misconception persists that loiasis would be cured as soon as the adult worm had disappeared from the eye besides the medical risks of these traditional treatments. Further investigations on traditional healer practices and their collaboration with the formal health sector should be carried out to broaden our understanding of healthcare seeking behaviour and to improve patient orientation towards adequate treatment modalities.

Our results showed a nuanced picture regarding response patterns of *L*. *loa* positive and negative participants. While the sample size may be too small for demonstration of conclusive differences, many dissimilarities may be explained by the personal experience of adult worm migration in patients suffering from migratory loiasis. Concordantly, migratory-loiasis patients were more often able to identify the parasite as a worm, associated *Chrysops* more often with the disease and knew better clinical symptoms associated with the eye worm migration. Finally, this subgroup stated more frequently to be prevented from work by loiasis, considered the disease to be more serious and estimated the risk of transmission to be higher than non-migratory participants did.

These findings are consistent with previous studies on distinct *L*. *loa* infection states and associated clinical and hematological manifestations. In this study it was shown that the history of eyeworm migration was associated with a wide range of clinical signs and symptoms, independent of the presence of microfilaremia. Neurological symptoms were notably found to be strongly associated with eye-worm-positive loiasis and not with microfilaremia itself. Also, in concordance with our results, exclusively microfilaremic individuals reported signs and symptoms in similar frequency as loiasis negative individuals [23]. Overall, our results show that people’s knowledge on the pathogen, signs and symptoms, the transmission of loiasis and their awareness of the risk of infection was augmented by having experienced an infection. Efforts in the future should therefore focus on informing affected communities in a preventive manner.

This study is not without limitations. As the first survey investigating the knowledge, attitudes and practices towards loiasis in a region of high transmission in rural Gabon, this sample size is limited and confined to one community. It is not known whether findings from this study can be directly extrapolated to other regions affected by loiasis. However, this setting allowed us to interview almost one fifth of the entire adult population leading to high level of internal validity. Moreover, the overall sample size compares well with previous KAP surveys on other neglected parasitic diseases in different regions of Africa and Asia [17,18,24,25]. Since our results align in most parts with these previously conducted studies, we believe that our findings can be generalized to most rural populations in the Central African region. However, further studies from different regions are needed.

## 5. Conclusion

Loiasis is a widely known infectious disease and is perceived as a health priority in this region of high *L*. *loa* transmission. Important gaps in knowledge concern foremost the mode of transmission of *L*. *loa*. Patients’ needs for diagnostics and treatment are largely unmet by the formal healthcare system. Overall, these data indicate the importance of loiasis for rural populations in high-transmission areas and provide guidance to ameliorate the services of the public health care system to meet the currently unmet needs in the management of loiasis.

## Supporting information

S1 Table Data that underlies this paper. (XLSX)
